# Delta-like ligand 4: A predictor of poor prognosis in clear cell renal cell carcinoma

**DOI:** 10.3892/ol.2014.2554

**Published:** 2014-09-24

**Authors:** WEI WANG, YI YU, YA WANG, XIAOMING LI, JUNSHENG BAO, GONGJIN WU, HONG CHANG, TINGKAI SHI, ZHONGJIN YUE

**Affiliations:** 1 Department of Urology, Institute of Urology, Gansu Nephro-Urological Clinical Center, Lanzhou, Gansu 730030, P.R. China; 2 Department of Gastroenterology, The Second Hospital of Lanzhou University, Lanzhou, Gansu 730030, P.R. China; 3 Department of Nephrology, The Second Hospital of Lanzhou University, Lanzhou, Gansu 730030, P.R. China; 4 Department of Pathology, The Second Hospital of Lanzhou University, Lanzhou, Gansu 730030, P.R. China

**Keywords:** delta-like ligand 4, clear cell renal cell carcinoma, survival, prognosis

## Abstract

Delta-like ligand 4 (Dll4)-Notch signaling is important in tumor angiogenesis; however, the prognostic value of D114 detection in patients with clear cell renal cell carcinoma (CCRCC) remains unclear. The present study aimed to determine whether the presence of high Dll4 expression levels was correlated with poor prognosis in CCRCC following curative resection. The D114 expression levels in four paired samples of CCRCC tissues and adjacent normal renal tissues were assayed by western blotting. Surgical specimens comprised 121 CCRCC tissue samples and 65 normal renal tissue samples, obtained from patients with CCRCC. The specimens were immunohistochemically assessed to determine Dll4 and vascular endothelial growth factor receptor 2 (VEGFR-2) expression levels. The prognostic significance of Dll4 expression levels was evaluated by the Kaplan-Meier method and Cox regression analysis. The correlation between Dll4 expression levels and VEGFR-2 expression levels, tumor stage, tumor grade and metastasis, was examined by χ^2^ test and multivariate logistic regression. As determined by the western blotting results, Dll4 protein expression levels were significantly increased in CCRCC tissues compared with those in adjacent non-cancerous tissues. From the analysis of the surgical specimens, 53 (43.8%) CCRCC patients exhibited immunohistochemically high Dll4 expression levels and 68 (56.2%) patients exhibited low Dll4 expression levels. The survival curves revealed that the patients with high Dll4 expression levels had significantly shorter survival times than the patients with low Dll4 expression levels (P<0.001). Multivariate survival analysis demonstrated that the presence of high Dll4 expression levels was independently associated with reduced overall survival and progression-free survival times (P=0.021 and 0.034, respectively). A positive correlation was also identified between Dll4 and VEGFR-2 expression levels (P=0.001). In conclusion, the results show that the presence of high Dll4 expression levels was clearly associated with high VEGFR-2 expression levels, tumor grade, tumor stage and poor prognosis in CCRCC patients. Therefore, inhibition of Dll4 may exert potent growth inhibitory effects on tumors resistant to anti-VEGF therapies for CCRCC.

## Introduction

Renal cell carcinoma (RCC) is the third most common urological cancer (1) and exhibits the highest mortality rate. Clear cell renal cell carcinoma (CCRCC) is the largest subtype of RCC and accounts for ~85% of RCC cases (2). Half of patients suffer from metastatic disease; the five-year survival rate for these patients is <10% and long-term remission is infrequent (3,4). Distant metastases and local recurrence are the main causes of fatalities following curative resection. Therefore, the identification of novel predictive and prognostic markers may result in targeted adjuvant therapies, and thus improve the prognosis in patients with early postoperative recurrence in CCRCC.

The vascular endothelial growth factor (VEGF) signaling pathway is important in tumor angiogenesis (5); inhibition of the pathway is currently a clinically approved and widely used therapy for cancer. Anti-VEGF therapy with bevacizumab has been shown to increase overall survival (OS) and/or progression-free survival (PFS) times in colorectal, breast and lung cancer patients (6). However, inherent or acquired resistance to anti-VEGF therapy is frequently observed in tumors (6), thus demonstrating the requirement for targeting additional angiogenesis pathways to fully exploit the strategies of anti-angiogenic cancer therapy. Notably, the Notch signaling pathway affects numerous biological processes, as well as cell fate determination, which has recently emerged as a critical regulator of developmental and tumor angiogenesis (7). In mammalian cells, Notch signaling mediators include five transmembrane Notch ligands [Jagged 1, Jagged 2, delta-like ligand 1 (Dll1), Dll3 and Dll4] and four Notch receptors (Notch 1–4) (8). Recently, Dll4 signaling through the corresponding Notch1 receptor has been identified as a critical component of physiological and pathological neovascularization (9). Dll4 is specifically induced by VEGF and functions as a negative angiogenesis regulator downstream of VEGF (10,11). Consistent with these findings, suppression of Dll4 inhibits tumor growth by promoting excessive and non-productive angiogenesis, even in tumors resistant to anti-VEGF therapy (9,12). In humans, Dll4 expression has been identified in tumors from the kidney, bladder, colon, brain and breast (13–16). In CCRCC, Dll4 expression was confined to the vasculature and was detected at levels nine-fold higher than those in the normal kidney (13,17). Previous studies have confirmed that Dll4 expression is an independent indicator of poor prognosis in several types of human malignancy, including lung, breast, pancreatic and bladder cancer (14,15,18,19). However, to the best of our knowledge, no study has been conducted to evaluate the prognostic value of Dll4 expression levels in patients with CCRCC. Therefore, in the present study, the expression levels of Dll4 in CCRCC were investigated, and an initial evaluation was conducted to analyze whether the presence of high Dll4 expression levels was correlated with poor prognosis following curative resection. In addition, the associations among Dll4 expression levels, VEGF receptor-2 (VEGFR-2) expression levels and tumor progression in patients with CCRCC were assessed.

## Materials and methods

### Patients and specimens

The study procedure was approved by the ethics committee of The Second Hospital of Lanzhou University (Lanzhou, China). For western blotting, fresh tumor tissues (later verified as CCRCC) and adjacent normal renal tissues were obtained intra-operatively from four patients who underwent radical nephrectomy at the Department of Urology in The Second Hospital of Lanzhou University. The tissue samples were then snap-frozen in liquid nitrogen and stored at −80°C until analysis. In addition, 121 paraffin-embedded CCRCC specimens (57 male and 64 female) and 65 normal renal tissue specimens were obtained from patients with resectable CCRCC. All patients underwent nephrectomy (partial or radical) during hospitalization at the Department of Urology, The Second Hospital of Lanzhou University between January 1, 2001 and December 31, 2010. Any cases of other types of renal carcinoma were excluded from this study. Additional exclusion criteria were a history of another type of cancer, and preoperative radiation or chemotherapy. All potentially eligible patients were interviewed to obtain written informed consent prior to surgery. The tumor stage was determined using the 2009 TNM staging classification system (20). The tumor grade was determined using the Fuhrman classification system (well-differentiated, grades 1 and 2; moderately differentiated, grade 3; and poorly differentiated, grade 4) (21). Patient profiles are summarized in Table I.

### Western blot analysis

The four paired samples of CCRCC tissues and adjacent normal renal tissues were solubilized in lysis buffer on ice. All lysates were centrifuged at 4°C for 10 min and total proteins were extracted from the tissues by a Keygen total protein extraction kit (SJ-200501; Nanjing Keygen Biotech. Co., Ltd, Nanjing, China) according to the manufacturer’s instructions and stored in a −80°C freezer until further use. For western blotting, 80 μg protein was separated by SDS-PAGE and transferred to polyvinylidene difluoride (PVDF) membranes, which were then blocked in 5% fat-free milk at room temperature for 2 h. Following incubation with polyclonal rabbit anti-human Dll4 antibody (Ab7280; dilution 1:500; Abcam, Cambridge, UK) at 4°C overnight and three washes for 15 min in 1X Tris-buffered saline, the PVDF membrane was incubated with horseradish peroxidase (HRP)-conjugated goat anti-rabbit polyclonal secondary antibodies (Ab137913; dilution 1:5,000; Abcam) at room temperature for 1 h. Subsequently, the membranes were washed three times, for 5 min each, with 1X Tris-buffered saline and the chemiluminescent HRP substrate was added to the PVDF membrane. The membranes were then exposed to X-ray medical films (Carestream Health, Inc., Rochester, NY, USA) in the dark. Immunoreactive proteins were visualized using an enhanced chemiluminescence HRP substrate (Millipore, Billerica, MA, USA) and polyclonal rabbit anti-human β-actin antibody (Ab75186; dilution 1:1,000; Abcam) served as a control for protein loading.

### Immunohistochemistry

Immunohistochemistry was performed using standard techniques. All tissues were cut into 0.5×0.5×0.5 cm blocks, fixed in 10% formalin solution and dehydrated through graded alcohol (70–100%), for 1 h respectively. The tissues were then cleared in xylene for 1 h. Parrafin wax immersion and embedding were conducted at 54°C for 1 h, then the blocks were cooled to room temperature and subjected to serial sections. For microscopic observation, the paraffin-embedded tissue sections, 4 mm in size, were dewaxed in xylene and rehydrated in graded alcohol and stained using hematoxylin and eosin. Endogenous peroxidase was inhibited using 3% hydrogen peroxide. Antigen retrieval was performed by boiling the tissue sections in citrate buffer (pH 6.0) for 10 min. Non-specific protein binding was conducted by incubating the sections with goat serum (Hyclone, Logan, UT, USA) for 30 min incubations. These treatments were alternated with rinses in phosphate-buffered saline (PBS). The sections were then incubated with primary antibodies against Dll4 (rabbit polyclonal; Ab7280; dilution 1:100; Abcam) and VEGFR-2 (rabbit monoclonal; 55B11; dilution 1:100; Cell Signaling Technology, Inc., Danvers, MA, USA) overnight at 4°C, respectively, washed three times in PBS, and incubated with secondary goat anti-rabbit polyclonal antibody (Ab137913; dilution 1:5,000; Abcam) conjugated to horseradish peroxidase (pv6001) for 30 min at room temperature. The membranes were washed again with PBS and then incubated with 3,3′-diaminobenzidine (dilution 1:20) stain, followed by counterstaining with hematoxylin blue. Non-neoplastic renal tissues from the same sample served as a control, aiming to omit the primary antibody in all cases.

### Immunostaining evaluation

The slides were independently evaluated under a Leica DMLP light microscope equipped with a Leica DFC camera (Leica Mikrosysteme Vertrieb GmbH, Wetzlar, Germany) by two pathologists (Professors Li and Shi) with no prior knowledge of the clinical data. Dll4 expression was localized to the tumor vasculature. For tumor vasculature, the brown particles were considered to signify positive cells. A semiquantitative scoring system was developed to evaluate staining intensity (0, negative; 1, weak; 2, moderate and 3, strong) and the percentage of positive cells (0, 0% cells; 1, ≤25% positive cells; 2, 26–50% positive cells and 3, >50% positive cells). In each slide, three fields were evaluated and the two scores were added: Low expression was designated as a total score of 0–3 and high expression as a total score of 4–6. The tumors were thus subdivided according to the protein expression levels in the different groups.

### Statistical analysis

The associations between Dll4 expression levels and CCRCC clinicopathological features were assessed by the χ^2^ test. The OS time period was measured as the time period between the date of surgery and the date the patient succumbed to disease or the date of the final follow-up. The PFS time period was measured as the time period between the date of surgery and either the date of disease relapse, the date the patient succumbed to disease or the date of the final follow-up. OS and PFS curves were calculated using the Kaplan-Meier method and compared by the log-rank test. The Cox regression model was used for multivariate analysis. All statistical analyses were performed using the SPSS version 18.0 statistical software package (SPSS, Inc., Chicago, IL, USA) and the quantification of the western blot bands was performed using Image J software (NIH, Bethesda, MD, USA). P<0.05 was considered to indicate a statistically significant difference.

## Results

### Patient characteristics

A total of 121 CCRCC patients (males, 57; females, 64) aged between 38 and 84 years (mean, 62 years) were included in the present study. According to the pathological tumor grading classification, 22 patients were considered to have poorly differentiated tumors, 31 to have moderately differentiated tumors and 68 to have well-differentiated tumors. With regard to the T-staging of the tumors, 54 tumors were considered as T1, 28 as T2, 29 as T3 and 10 as T4. The mean follow-up duration at the time of analysis was 48.6 months (range, 6–96 months). A total of 29 metastatic CCRCC cases and 92 non-metastatic cases were identified. Demographic and clinicopathological variables within the cohort are summarized in Table I.

### Dll4 expression levels in CCRCC specimens and non-cancerous tissues

**Figure 1:**
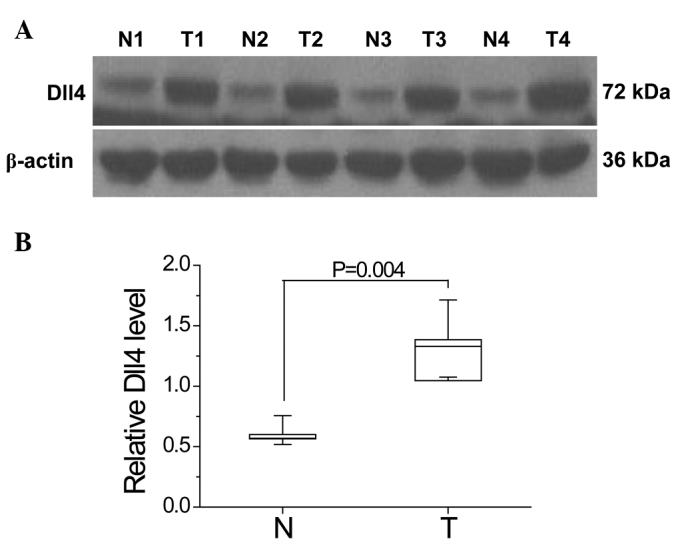
Expression levels of delta-like ligand 4 (Dll4) protein in clear cell renal cell carcinoma (CCRCC; T) and non-cancerous tissues (N), assayed by western blotting. (A) Western blot analysis reveals higher Dll4 expression levels in CCRCC than those in non-cancerous tissues. (B) Quantification of the bands in Fig. 1a using ImageJ software. All Dll4 expression levels were normalized to those of the corresponding internal control (β-actin).

Western blot analysis was performed to analyze the differences in Dll4 expression levels between CCRCC and non-cancerous tissues in four CCRCC patients who had undergone radical nephrectomy. The results show that Dll4 expression levels were significantly increased in the CCRCC tissues compared with the non-cancerous tissues (P=0.004; Fig. 1).

**Figure 2:**
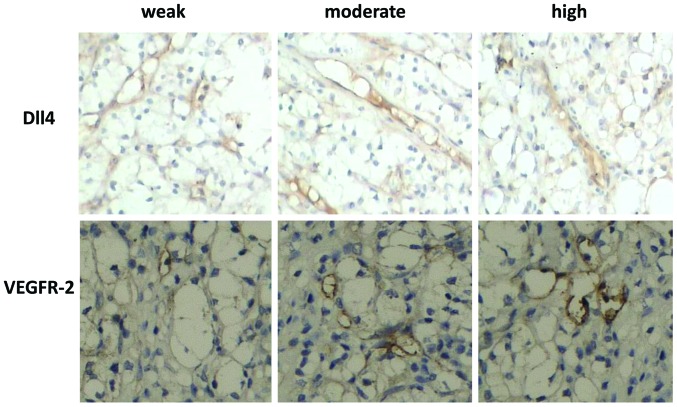
Immunohistochemical staining for delta-like ligand 4 (Dll4) and vascular endothelial growth factor receptor 2 (VEGFR-2) in clear cell renal cell carcinoma (CCRCC). Dll4 was mainly expressed in the tumor vasculature, and VEGFR-2 was mainly expressed in the tumor vasculature and the cytoplasm. Weak Dll4 expression (score 1), moderate Dll4 expression (score 4) and (high Dll4 expression (score 6) in CCRCC. Weak VEGFR-2 expression (score 1), moderate VEGFR-2 expression (score 4) and high VEGFR-2 expression (score 6) in CCRCC. Original magnification, ×200.

Immunohistochemical analysis was performed to demonstrate the presence and location of Dll4 in 121 CCRCC and 65 non-cancerous tissue specimens from CCRCC patients. As shown in Fig. 2, Dll4 and VEGFR-2 were localized in the blood vessels in CCRCC. Significant increases in Dll4 expression levels were observed in CCRCC tissues compared with those of non-cancerous tissues (P<0.001, Table II).

### Dll4 expression levels and CCRCC features

The associations between Dll4 expression levels and CCRCC clinicolpathological variables are summarized in Table I. No significant association between Dll4 expression levels and patient age (P=0.538), gender (P=0.276) or tumor size (P=0.782) was detected. However, Dll4 was found to be significantly correlated with tumor metastasis (P=0.012), tumor T-stage (P=0.023), tumor grade (P=0.033) and VEGFR-2 expression levels (P=0.001). High Dll4 and VEGFR-2 expression levels were observed in 53 (43.8%) and 75 (62.0%) patients, respectively. These findings imply that high Dll4 expression levels are associated with tumor differentiation, invasion, metastasis and angiogenesis, which in turn are associated with tumorigenesis and tumor progression.

### Association between Dll4 expression levels and CCRCC prognosis

**Figure 3:**
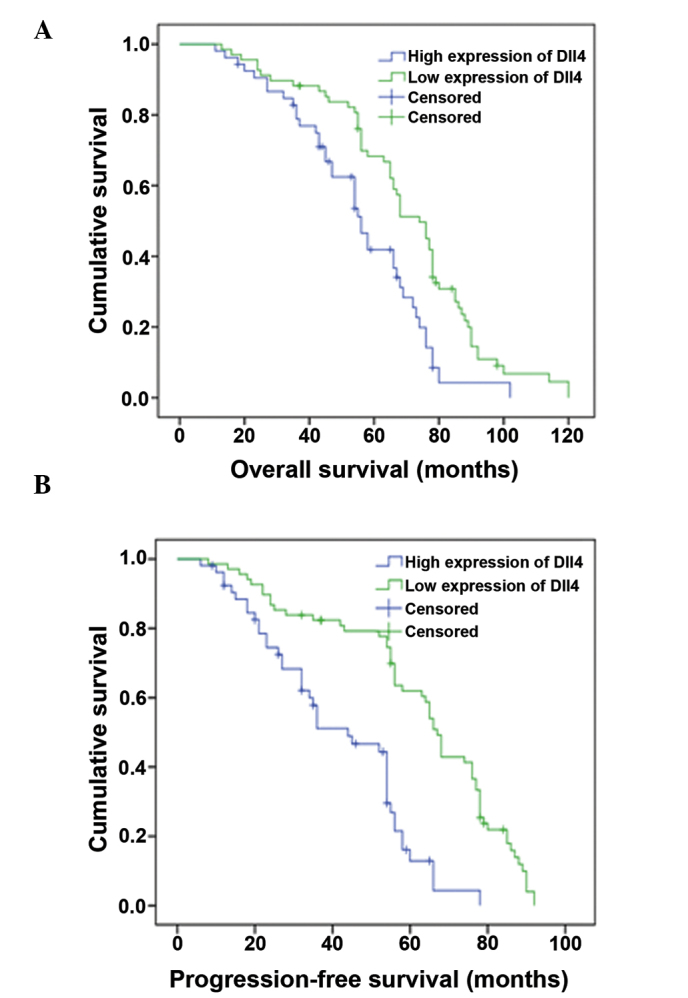
Kaplan-Meier estimates of cumulative overall survival and progression-free survival times, according to delta-like ligand 4 (Dll4) expression levels in clear cell renal cell carcinoma groups. (A) Significant differences in overall survival times were observed between the high and low Dll4 expression subgroups (P<0.001). (B) Significant differences in progression-free survival were observed between the high and low Dll4 expression subgroups (P<0.001).

The correlations between Dll4 expression levels and OS and PFS parameters were evaluated by Kaplan-Meier survival analysis with log-rank statistic for determining significance. As shown in Fig. 3, the patients with high Dll4 expression levels had significantly worse OS (P<0.001) and PFS (P<0.001) times than those with low Dll4 expression levels (mean OS times, 38.9 and 52.9 months, respectively; mean PFS times, 21.5 and 33.7 months, respectively). In a multivariate OS and PFS analysis determined by the Cox proportional-hazards regression model, the independent predictive value for Dll4 expression levels, as well as relevant clinical and pathological parameters, including age, gender, tumor size, the presence of metastases, T-stage, T-grade and VEGFR-2 expression levels, were detected. As shown in Tables III and IV, an increased level of Dll4 expression was shown to be an independent prognostic predictor of OS (P=0.021) and PFS (P=0.034) times, in addition to the presence of tumor metastasis (OS, P=0.001), tumor stage, tumor grade (OS, P=0.045) and high expression levels of VEGFR-2. (OS, P=0.018).

## Discussion

RCC is an intractable disease due to inherent tumor resistance to chemotherapy and radiotherapy (22). A previous study demonstrated that the VEGF signaling pathway is important in angiogenesis in renal cancer (5). Experimental systems have shown that VEGF signaling induces Dll4 expression, which acts as a negative feedback regulator to restrain vascular sprouting and branching (10,11,23). Functional tumor angiogenesis requires a balance of VEGF signaling and Dll4 expression (9,12,24). D114 expression has been detected at nine-fold higher levels in CCRCC than in the normal kidney, with expression confined to the vasculature (13,17). In the present study, Dll4 protein was detected in fresh paired human CCRCC and non-cancerous tissues through western blotting, and the expression levels of Dll4 protein in 121 paraffin-embedded CCRCC specimens and 65 normal renal tissues were also examined using immunohistochemistry. The results revealed that Dll4 expression levels were higher in CCRCC than those in the corresponding non-cancerous tissues. Furthermore, Dll4 expression levels were found to be associated with tumor differentiation, invasion and metastasis. Compared with the primary Dll4 tissues, metastatic CCRCC tissues exhibited more frequent Dll4 overexpression, which confirms that the presence of metastasis predicts an inferior clinical outcome. Increased Dll4 expression levels were clearly correlated with increased VEGFR-2 expression levels, which was consistent with results from previous studies (9–11,24). All findings suggest that Dll4 expression is key in tumor angiogenesis, which indicates that D114 overexpression may be associated with poor prognosis in human cancer.

In the examination of prognostic factors, the results from the Kaplan-Meier survival analysis with log-rank statistic suggest that the presence of high Dll4 expression levels was positively correlated with reduced OS and PFS times. In multivariate analysis, TNM stage, grade, tumor size, age and gender were analyzed, as well as expression levels of Dll4, using the Cox regression model. The results revealed that the level of Dll4 expression was an independent prognostic factor for OS and PFS times. Previous studies observed that Dll4 expression was an independent indicator of poor prognosis in several types of human cancer, including lung, breast, bladder and nasopharyngeal cancer (14,15,18,25). The present study extended this finding to CCRCC. A markedly worse prognosis was observed in patients with high Dll4 expression levels than in those with low Dll4 expression levels. Furthermore, inhibition of Dll4-Notch has been shown to result in tumor growth inhibition associated with the formation of a non-functional, hypersprouting tumor vasculature (9,26,27). Thus, the Dll4/Notch signaling pathway activation loop appears to promote tumor formation and progression.

In recent years, anti-angiogenesis-targeted therapies have been identified as promising therapeutic strategies. Certain newly developed agents that target the VEGF signaling pathway, such as the small-molecular VEGF receptor inhibitors, sorafenib and sunitinib, and the anti-VEGF monoclonal antibody bevacizumab, have shown encouraging treatment benefits in advanced RCC patients in randomized controlled trials (28–30), but the effects are variable and incremental, and acquired or innate resistance is frequently encountered (31,32). Anti-VEGF therapy acts to prune vascular sprouts and reduce new vessel growth (27,33), which is in contrast to the abovementioned cellular effects of inhibiting the Dll4-Notch signaling pathway. Notably, preclinical studies have demonstrated that Dll4 suppression exerts strong growth inhibitory effects on tumors that are resistant to anti-VEGF therapies (9,26). In addition, simultaneously targeting Dll4 and VEGF has been shown to generate additive antitumor effects compared with single agents in numerous tumor models (34). Compared with Dll4 expression levels in lung, breast, pancreatic and bladder cancer, Dll4 expression in CCRCC has been detected at levels nine-fold higher than those in the normal kidney (13,17). Therefore, CCRCC patients may benefit most from the therapeutic targeting of angiogenesis by simultaneous inhibition of the Dll4-Notch and VEGF signaling pathways.

Thus far, few CCRCC-specific biomarkers have been used in the clinical setting for diagnosis and prognosis. Although Dll4 appears not to be a specific marker of CCRCC, patients may benefit due to the significance of D114 in determining patient prognosis. From a clinical viewpoint, the presence of high Dll4 expression levels may be considered a risk factor for tumor progression; therefore, implementing a strict systemic therapeutic plan, such as immunotherapy, angiogenesis inhibitor drugs or chemotherapy, following surgery, along with regular investigation may improve prognosis.

In conclusion, high Dll4 expression levels were demonstrated to be clearly associated with high VEGFR-2 expression levels, indicators of pathological aggressiveness (such as metastasis, pathological grade and tumor stage) and poor prognosis in CCRCC patients. Therefore, inhibition of Dll4 may exert potent growth inhibitory effects on CCRCC tumors that are resistant to anti-VEGF therapies.

## Figures and Tables

**Table I:** Correlation between Dll4 expression levels and clinicopathological factors.

		Dll4 expression levels		
			
Parameters	N	High (53)	Low (68)	P-value
Age				
≤Mean	70	29	41	0.538
>Mean	51	24	27	
Gender				
Male	57	22	35	0.276
Female	64	31	33	
Size, mm				
≤70	77	33	44	0.782
>70	44	20	24	
Metastasis				
Metastasis	29	18	11	0.012
No metastasis	92	35	57	
T-stage^a^				
T1+T2	82	31	51	0.023
T3+T4	39	22	17	
Grade^b^				
1+2	68	24	44	0.033
3+4	53	29	24	
VEGFR-2				
High	75	42	33	0.001
Low	46	11	35	

a Tumor stage was determined using the 2009 TNM staging classification system (20);

b tumor grade was determined using the Fuhrman classification system (well-differentiated, grades 1 and 2; moderately differentiated, grade 3; and poorly differentiated, grade 4) (21).

D114, delta-like ligand 4; VEGFR-2, vascular endothelial growth factor receptor 2.

**Table II:** Dll4 expression levels in normal renal tissues and CCRCC.

		Dll-4 expression levels		
			
Variable	n	High	Low	P-value
CCRCC	121	53	68	<0.001
NRT	65	9	56	

D114, delta-like ligand 4; CCRCC, clear cell renal cell carcinoma; NRT, normal renal tissue.

**Table III:** Cox regression model analysis of overall survival times.

Variable	β	Standard error	Wald	df	P-value	Exp (β)
Age				1	0.874	
Gender				1	0.231	
Tumor size				1	0.064	
Tumor stage	0.874	0.452	4.487	1	0.006	2.457
Tumor grade	1.023	0.478	5.121	1	0.045	3.124
Metastasis	0.748	0.364	4.213	1	0.001	4.215
High Dll4	0.645	0.412	5.456	1	0.021	2.741
High VEGFR-2	0.946	0.547	4.365	1	0.018	3.102

D114, delta-like ligand 4; VEGFR-2, vascular endothelial growth factor receptor 2.

**Table IV:** Cox regression model analysis of progression-free survival times.

Variable	β	Standard error	Wald	df	P-value	Exp (β)
Age				1	0.874	
Gender				1	0.231	
Tumor size				1	0.064	
Tumor stage	0.912	0.675	3.892	1	0.021	2.872
Tumor grade	1.235	0.364	4.768	1	0.025	3.432
Metastasis	0.872	0.392	7.113	1	0.008	4.214
High Dll4	0.587	0.454	5.821	1	0.034	3.231
High VEGFR-2	0.798	0.442	4.365	1	0.018	3.569

D114, delta-like ligand 4; VEGFR-2, vascular endothelial growth factor receptor 2.
